# Supplementary figures and images for: Antennal sensilla diversity in diurnal and nocturnal fireflies (Coleoptera, Lampyridae)
Source: PLoS One. 2025 Jun 12;20(6):e0323722. doi: 10.1371/journal.pone.0323722 (PMC12161595; doi:10.1371/journal.pone.0323722)

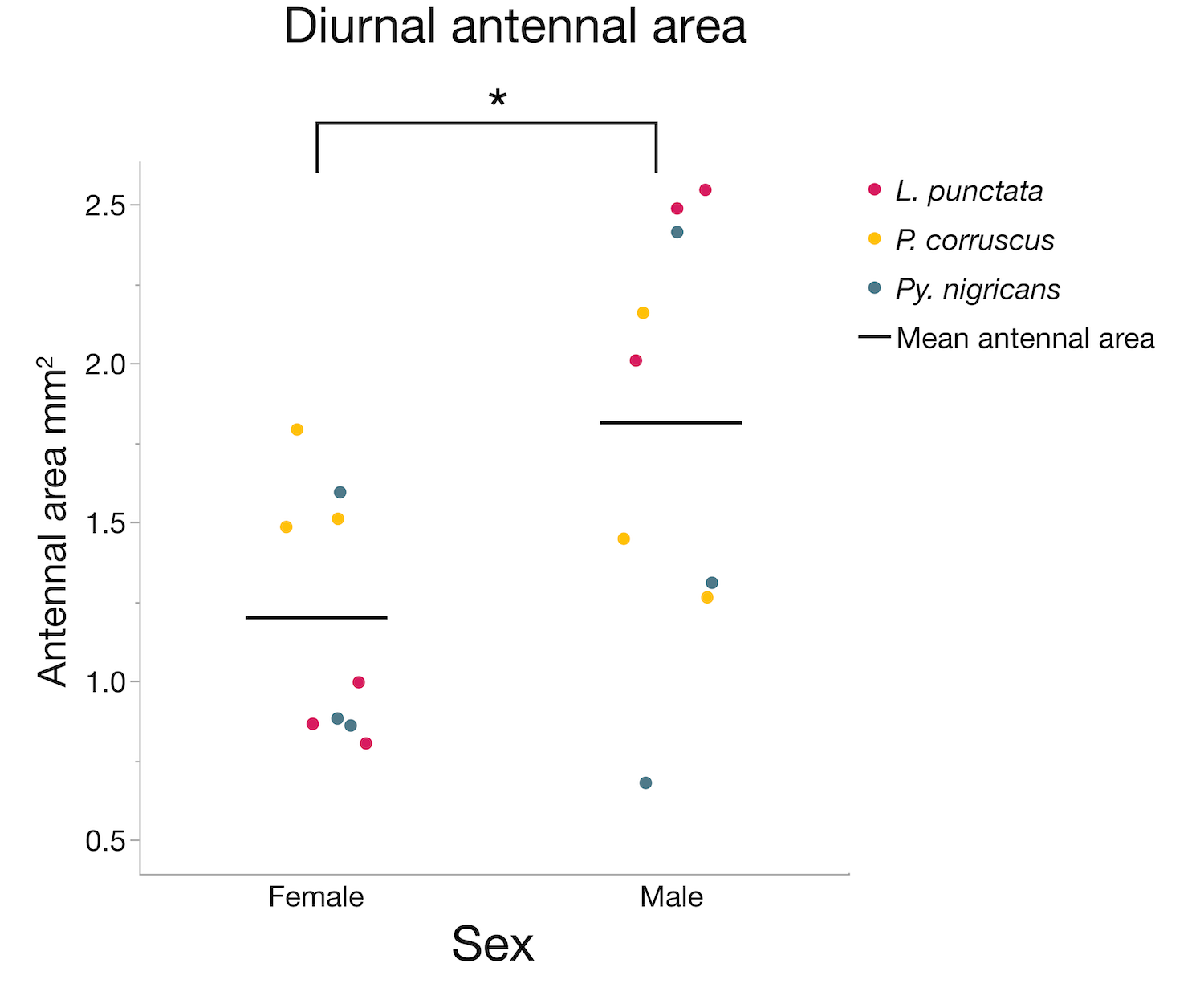

Supplement: Figure S1 — (A) In diurnal species, males have significantly larger antennal areas than females (x̄Male = 1.812 ± 0.66 mm2, x̄Female = 1.2 ± 0.38 mm2; Student’s t ratio = -3.86, p = 0.0005). (TIF) [file pone.0323722.s001.tif]

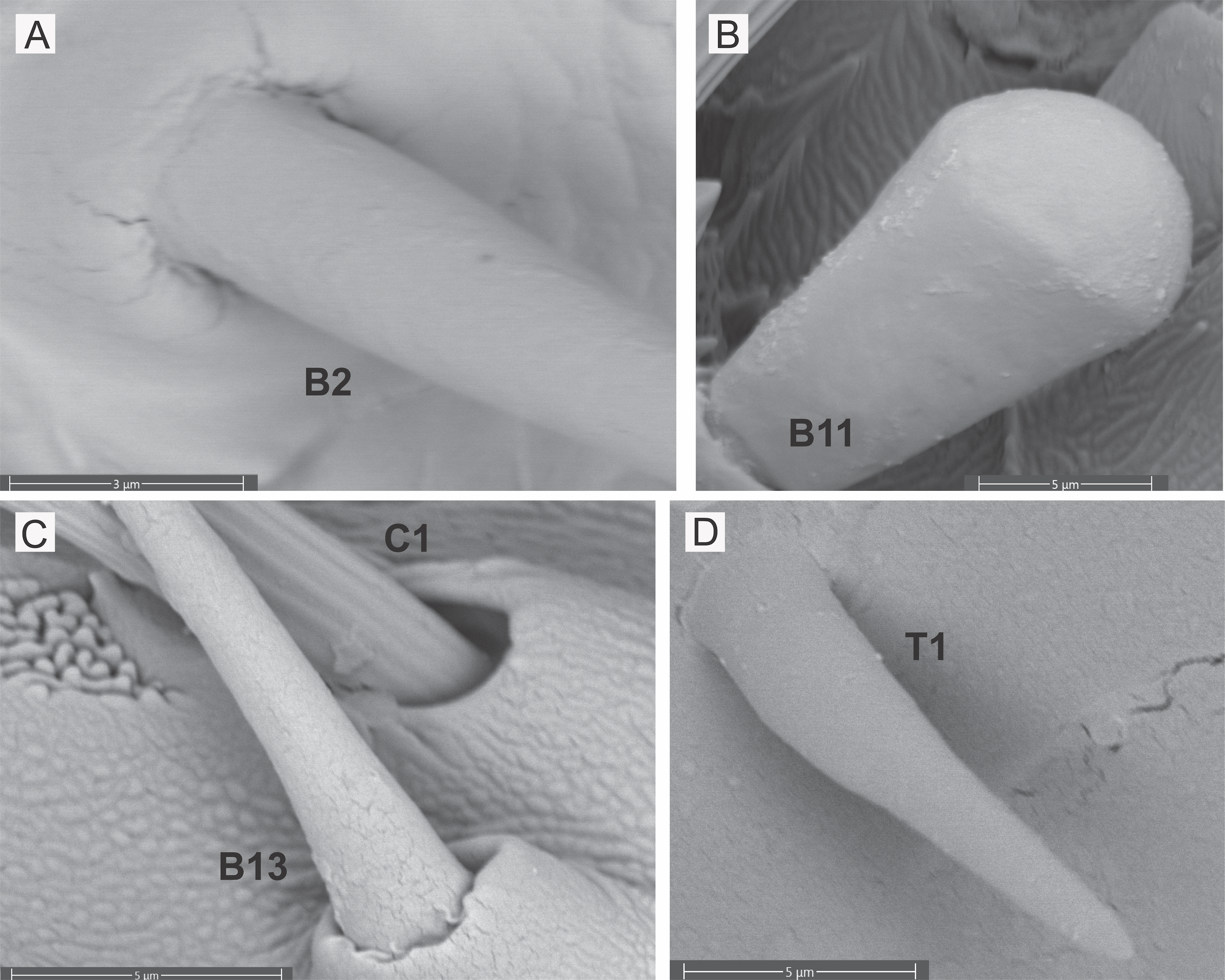

Supplement: Figure S2 — (TIF) [file pone.0323722.s002.tif]
