# Supplementary material for: Antennal sensilla diversity in diurnal and nocturnal fireflies (Coleoptera, Lampyridae)
Source: PLoS One. 2025 Jun 12;20(6):e0323722. doi: 10.1371/journal.pone.0323722 (PMC12161595; doi:10.1371/journal.pone.0323722)
Supplement: Table S1 — Species name, specimen number, KSH voucher ID number, and sex. (DOCX) [file pone.0323722.s010.docx]

**Table S1. Voucher specimens used for SEM imaging.**

| Species | Specimen number | KSH ID number | Sex |
| --- | --- | --- | --- |
| *Lucidota punctata* | LpF1 | KSH5445 | Female |
| *Lucidota punctata* | LpF2 | KSH1397 | Female |
| *Lucidota punctata* | LpF3 | KSH5446 | Female |
| *Lucidota punctata* | LpM1 | KSH5509 | Male |
| *Lucidota punctata* | LpM2 | KSH5507 | Male |
| *Lucidota punctata* | LpM3 | KSH5902 | Male |
| Luciolinae sp. | LucF1 | KSHX1466 | Female |
| Luciolinae sp. | LucF2 | KSHX1467 | Female |
| Luciolinae sp. | LucF3 | KSHX1468 | Female |
| Luciolinae sp. | LucM2 | KSHX1463 | Male |
| Luciolinae sp. | LucM3 | KSHX1464 | Male |
| Luciolinae sp. | LucM4 | KSHX1465 | Male |
| *Phausis christineae* | PcF3 | KSH4134 | Female |
| *Phausis christineae* | PcF4 | KSH4133 | Female |
| *Phausis christineae* | PcF5 | KSH4132 | Female |
| *Phausis christineae* | PcM1 | KSH4106 | Male |
| *Phausis christineae* | PcM2 | KSH4109 | Male |
| *Phausis christineae* | PcM3 | KSH44137 | Male |
| *Photinus corruscus* | EcF1 | KSH5533 | Female |
| *Photinus corruscus* | EcF2 | KSH5348 | Female |
| *Photinus corruscus* | EcF3 | KSH5440 | Female |
| *Photinus corruscus* | EcM1 | KSH5532 | Male |
| *Photinus corruscus* | EcM2 | KSH5537 | Male |
| *Photinus corruscus* | EcM3 | KSH5538 | Male |
| *Photinus pyralis* | PpF1 | KSH91 | Female |
| *Photinus pyralis* | PpF2 | KSH5901 | Female |
| *Photinus pyralis* | PpF3 | KSH4259.2 | Female |
| *Photinus pyralis* | PpM1 | KSH844 | Male |
| *Photinus pyralis* | PpM2 | KSH4235 | Male |
| *Photinus pyralis* | PpM3 | KSH4259.1 | Male |
| *Photuris lucicrescens* | PlF1 | KSH1714 | Female |
| *Photuris lucicrescens* | PlF2 | KSH1712 | Female |
| *Photuris lucicrescens* | PlF3 | KSH1713 | Female |
| *Photuris lucicrescens* | PlM1 | KSH1472 | Male |
| *Photuris lucicrescens* | PlM2 | KSH1473 | Male |
| *Photuris lucicrescens* | PlM3 | KSH1475 | Male |
| *Pyropyga nigricans* | PnF1 | KSH335 | Female |
| *Pyropyga nigricans* | PnF2 | KSH1422 | Female |
| *Pyropyga nigricans* | PnF3 | KSH445 | Female |
| *Pyropyga nigricans* | PnM1 | KSH336 | Male |
| *Pyropyga nigricans* | PnM2 | KSH1432 | Male |
| *Pyropyga nigricans* | PnM3 | KSH44 | Male |
