# Supplementary material for: Antennal sensilla diversity in diurnal and nocturnal fireflies (Coleoptera, Lampyridae)
Source: PLoS One. 2025 Jun 12;20(6):e0323722. doi: 10.1371/journal.pone.0323722 (PMC12161595; doi:10.1371/journal.pone.0323722)
Supplement: Table S2 — Individual mechanosensilla (C1, C2, SC) counts (mean ± stdev) for each species (F: 3 females, M: 3 males, D: diurnal, N: Nocturnal, L. = Lucidota, P. = Photinus, Py. = Pyropyga, Pha. = Phausis, Ph. = Photuris). (DOCX) [file pone.0323722.s011.docx]

**Table S2. Mechanosensilla counts.**

| Species | Sex | Activity | C1 (N) | C2 (N) | SC (N) |
| --- | --- | --- | --- | --- | --- |
| *L. punctata* | F | D | 2082 ± 154 | 43 ± 12 | 8 ± 7 |
|  | M | D | 3351 ± 236 | 54 ± 12 | 0 |
| *P. corruscus* | F | D | 442 7± 555 | 54 ± 15 | 1 ± 1 |
|  | M | D | 4464 ± 435 | 66 ± 21 | 1 ± 1 |
| *Py. nigricans* | F | D | 3599 ± 843 | 49 ± 22 | 17 ± 10 |
|  | M | D | 2732 ± 664 | 67 ± 7 | 1 ± 1 |
| Luciolinae sp. | F | N | 1622 ± 137 | 51 ± 10 | 3 |
|  | M | N | 1721 ± 124 | 67 ± 6 | 1 ± 1 |
| *Pha. christineae* | F | N | 36 ± 3 | 2 ± 1 | 0 |
|  | M | N | 525 ± 103 | 33 ± 12 | 0 |
| *P. pyralis* | F | N | 2892 ± 175 | 82 ± 14 | 23 ± 6 |
|  | M | N | 2935 ± 296 | 74 ± 19 | 24 ± 4 |
| *Ph. lucicrescens* | F | N | 1860 ± 112 | 87 ± 7 | 0 |
|  | M | N | 1895 ± 1492 | 92 ± 22 | 1 ± 2 |

Individual mechanosensilla (C1, C2, SC; C: sensilla chaetica, SC: sensilla campaniform) counts (mean ± stdev) for each species (F: 3 females, M: 3 males, D: diurnal, N: Nocturnal, *L.* = *Lucidota*, *P.* = *Photinus*, *Py.* = *Pyropyga*, *Pha. = Phausis*, *Ph. = Photuris*).
