# Supplementary material for: Antennal sensilla diversity in diurnal and nocturnal fireflies (Coleoptera, Lampyridae)
Source: PLoS One. 2025 Jun 12;20(6):e0323722. doi: 10.1371/journal.pone.0323722 (PMC12161595; doi:10.1371/journal.pone.0323722)
Supplement: Table S3 — Individual chemosensilla (B1-B3, B7, B10-B13, T1) counts (mean ± stdev) for each species (F: 3 females, M: 3 males, D: diurnal, N: Nocturnal, L. = Lucidota, P. = Photinus, Py. = Pyropyga, Pha. = Phausis, Ph. = Photuris). (DOCX) [file pone.0323722.s012.docx]

**Table S3. Chemosensilla counts.**

| Species | Sex | Active | B1 (N) | B2 (N) | B3 (N) | B7 (N) | B10 (N) | B11 (N) | B12 | B13 | T1 (N) |
| --- | --- | --- | --- | --- | --- | --- | --- | --- | --- | --- | --- |
| *L. punctata* | F | D | 0 | 0 | 0 | 23 ± 18 | 350 ± 31 | 29 ± 13 | 0 | 0 | 0 |
|  | M | D | 0 | 0 | 0 | 35 ± 23 | 506 ± 64 | 2881 ± 139 | 0 | 0 | 0 |
| *P. corruscus* | F | D | 191± 101 | 295 ± 77 | 267 ± 53 | 4 ± 4 | 0 | 0 | 0 | 0 | 0 |
|  | M | D | 410 ± 141 | 185 ± 26 | 206 ± 51 | 29 ± 9 | 0 | 0 | 0 | 0 | 0 |
| *Py. nigricans* | F | D | 32 ± 19 | 0 | 755 ± 303 | 35 ± 14 | 0 | 0 | 0 | 0 | 0 |
|  | M | D | 15 ± 12 | 0 | 1151 ± 478 | 12 ± 10 | 0 | 0 | 0 | 0 | 0 |
| Luciolinae sp. | F | N | 0 | 0 | 0 | 27 ± 30 | 0 | 0 | 0 | 442±29 | 0 |
|  | M | N | 0 | 0 | 0 | 29 ± 2 | 0 | 0 | 13±3 | 488±29 | 0 |
| *Pha. christineae* | F | N | 0 | 0 | 0 | 0 | 0 | 0 | 0 | 0 | 10 ± 4 |
|  | M | N | 0 | 0 | 0 | 0 | 0 | 0 | 0 | 0 | 572 ± 24 |
| *P. pyralis* | F | N | 482 ± 90 | 0 | 207 ± 10 | 21 ± 6 | 0 | 0 | 0 | 0 | 0 |
|  | M | N | 622 ± 90 | 0 | 269 ± 103 | 12 ± 14 | 0 | 0 | 0 | 0 | 0 |
| *Ph. lucicrescens* | F | N | 0 | 825 ± 52 | 0 | 15 ± 5 | 0 | 0 | 0 | 0 | 0 |
|  | M | N | 0 | 704 ± 252 | 0 | 17 ± 19 | 0 | 0 | 0 | 0 | 0 |

Individual chemosensilla (B1-B3, B7, B10-B13, T1; B: sensilla basiconica, T: sensilla trichoidea) counts (mean ± stdev) for each species (F: 3 females, M: 3 males, D: diurnal, N: Nocturnal, *L.* = *Lucidota*, *P.* = *Photinus*, *Py.* = *Pyropyga*, *Pha. = Phausis*, *Ph. = Photuris*).
