# Supplementary material for: Antennal sensilla diversity in diurnal and nocturnal fireflies (Coleoptera, Lampyridae)
Source: PLoS One. 2025 Jun 12;20(6):e0323722. doi: 10.1371/journal.pone.0323722 (PMC12161595; doi:10.1371/journal.pone.0323722)
Supplement: Table S4 — Individual mechanosensilla (C1, C2, SC) density (mean ± stdev N/mm2) of each species (F: 3 females, M: 3 males, D: diurnal, N: Nocturnal, L. = Lucidota, P. = Photinus, Py. = Pyropyga, Pha. = Phausis, Ph. = Photuris). (-) type absent. (DOCX) [file pone.0323722.s013.docx]

**Table S4. Mechanosensilla densities.**

| Species | Sex | Activity | C1 (N/mm^2^) | C2 (N/mm^2^) | SC (N/mm^2^) |
| --- | --- | --- | --- | --- | --- |
| *L. punctata* | F | D | 2373 ± 386 | 49 ± 14 | 10 ± 9 |
|  | M | D | 1443 ± 200 | 23 ± 8 | - |
| *P. corruscus* | F | D | 2776 ± 203 | 34 ± 8 | 0.4 ± 0.8 |
|  | M | D | 2850 ± 512 | 401 ± 12 | 0.5± 0.5 |
| *Py. nigricans* | F | D | 3354 ± 592 | 51 ± 33 | 15 ± 10 |
|  | M | D | 2376 ± 1287 | 58 ± 31 | 1 ± 0.9 |
| Luciolinae sp*.* | F | N | 2970 ± 101 | 93 ± 30 | 5 ± 6 |
|  | M | N | 3747 ± 95 | 147 ± 21 | 2 ± 3 |
| *Pha. christineae* | F | N | 200 ± 57 | 128 ±25 | - |
|  | M | N | 2728 ± 170 | 169 ± 26 | - |
| *P. pyralis* | F | N | 1474 ± 324 | 41 ± 8 | 11 ± 2 |
|  | M | N | 1195 ± 19 | 30 ± 8 | 10 ± 2 |
| *Ph. lucicrescens* | F | N | 727 ± 153 | 33 ± 5 | - |
|  | M | N | 983 ± 41 | 47 ± 8 | 0.4 ± 0.7 |

Individual mechanosensilla (C1, C2, SC: C: sensilla chaetica, SC: sensilla campaniform) density (mean ± stdev N/mm^2^) of each species (F: 3 females, M: 3 males, D: diurnal, N: Nocturnal, *L.* = *Lucidota*, *P.* = *Photinus*, *Py.* = *Pyropyga*, *Pha. = Phausis*, *Ph. = Photuris*; (-) type absent).
