# Supplementary material for: Antennal sensilla diversity in diurnal and nocturnal fireflies (Coleoptera, Lampyridae)
Source: PLoS One. 2025 Jun 12;20(6):e0323722. doi: 10.1371/journal.pone.0323722 (PMC12161595; doi:10.1371/journal.pone.0323722)
Supplement: Table S5 — Individual chemoreceptor (B1-B3, B7, B10-B13, T1) density (mean ± stdev) of each species (F: 3 females, M: 3 males, D: diurnal, N: nocturnal, L. = Lucidota, P. = Photinus, Py. = Pyropyga, Pha. = Phausis, Ph. = Photuris). (-) type absent. (DOCX) [file pone.0323722.s014.docx]

**Table S5.** **Chemosensilla densities.**

| Species | Sex | Active | B1 (N/mm^2^) | B2 (N/mm^2^) | B3 (N/mm^2^) | B7 (N/mm^2^) | B10 (N/mm^2^) | B11 (N/mm^2^) | T1 (N/mm^2^) |
| --- | --- | --- | --- | --- | --- | --- | --- | --- | --- |
| *L. punctata* | F | D | - | - | - | 28 ± 23 | 396 ± 27 | 34 ± 18 | - |
|  | M | D | - | - | - | 15 ± 9 | 216 ± 8 | 1245 ± 203 | - |
| *P. corruscus* | F | D | 122 ± 70 | 188 ± 61 | 171 ± 48 | 3 ± 3 | - | - | - |
|  | M | D | 253 ± 43 | 123 ± 47 | 131 ± 35 | 18 ± 5 | - | - | - |
| *Py. nigricans* | F | D | 28 ± 15 | - | 797 ± 473 | 31 ± 4 | - | - | - |
|  | M | D | 9 ± 3 | - | 980 ± 530 | 13 ± 17 | - | - | - |
| Luciolinae sp. | F | N | - | - | - | 49 ± 53 | - | - | - |
|  | M | N | - | - | - | 63 ± 7 | - | - | - |
| *Pha. christineae* | F | N | - | - | - | - | - | - | 128 ± 25 |
|  | M | N | - | - | - | - | - | - | 170 ± 26 |
| *P. pyralis* | F | N | 244 ± 61 | - | 106 ± 27 | 10 ± 2 | - | - | - |
|  | M | N | 256 ± 51 | - | 108 ± 34 | 5 ± 5 | - | - | - |
| *Ph. lucicrescens* | F | N | - | 323 ± 74 | - | 6 ± 3 | - | - | - |
|  | M | N | - | 360 ± 95 | - | 9 ± 9 | - | - | - |

Individual chemoreceptor (B1-B3, B7, B10-B13, T1; B: sensilla basiconica, T: sensilla trichoidea) density (mean ± stdev) of each species (F: 3 females, M: 3 males, D: diurnal, N: nocturnal, *L.* = *Lucidota*, *P.* = *Photinus*, *Py.* = *Pyropyga*, *Pha. = Phausis*, *Ph. = Photuris*). (-) type absent.
