# Supplementary material for: Antennal sensilla diversity in diurnal and nocturnal fireflies (Coleoptera, Lampyridae)
Source: PLoS One. 2025 Jun 12;20(6):e0323722. doi: 10.1371/journal.pone.0323722 (PMC12161595; doi:10.1371/journal.pone.0323722)
Supplement: Table S6 — Mean counts and densities (mean ± standard deviation) for X1 sensilla by species and sex. *X1 sensilla were not present in all specimens within a species, specimens lacking these sensilla were excluded from mean and standard deviation calculations. (F: 3 females, M: 3 males, D: diurnal, N: nocturnal, L. = Lucidota, P. = Photinus, Py. = Pyropyga, Pha. = Phausis, Ph. = Photuris). (-) type absent. (DOCX) [file pone.0323722.s015.docx]

**Table S6. X1 sensilla counts and densities.**

| Species | Sex | Active | X1 counts* | X1 density* | Sample size |
| --- | --- | --- | --- | --- | --- |
| *L. punctata* | F | D | 0 | - | - |
|  | M | D | 0 | - | - |
| *P. corruscus* | F | D | 22 | 15 | 1 |
|  | M | D | 13 ± 4 | 8 ± 3 | 3 |
| *Py. nigricans* | F | D | 4 ± 4 | 4 ± 5 | 2 |
|  | M | D | 5 | 7 | 1 |
| Luciolinae sp. | F | N | 0 | - | - |
|  | M | N | 0 | - | - |
| *Pha. christineae* | F | N | 0 | - | - |
|  | M | N | 0 | - | - |
| *P. pyralis* | F | N | 0 | - | - |
|  | M | N | 0 | - | - |
| *Ph. lucicrescens* | F | N | 16 | 7 | 1 |
|  | M | N | 4 | 2 | 1 |

Mean counts and densities (mean ± standard deviation) for X1 sensilla by species and sex (D: diurnal, N: nocturnal, *L.* = *Lucidota*, *P.* = *Photinus*, *Py.* = *Pyropyga*, *Pha. = Phausis*, *Ph. = Photuris*; (-) type absent). *X1 sensilla were not present in all specimens within a species, specimens lacking these sensilla were excluded from mean and standard deviation calculations.
